# Focal seizures during adrenocorticotropic hormone therapy in a school-aged boy: a case report

**DOI:** 10.1186/s13256-022-03429-0

**Published:** 2022-05-24

**Authors:** Yoshitaka Ota, Shuichi Shimakawa, Hikaru Tsuda-Kitahara, Miho Fukui, Mitsuru Kashiwagi, Akira Ashida

**Affiliations:** 1 Department of Pediatrics, Hirakata Municipal Hospital, Hirakata, Osaka Japan; 2 Department of Pediatrics, Osaka Medical and Pharmaceutical University Hospital, 2-7 Daigaku-machi, Takatsuki, Osaka 569-8686 Japan

**Keywords:** Hemiconvulsion–hemiplegia–epilepsy syndrome, Posttraumatic epilepsy, ACTH treatment, ACTH-induced seizures

## Abstract

**Background:**

Adrenocorticotropic hormone therapy for infantile spasms, including West syndrome, has been previously reported to induce seizures. We present the findings for a school-aged child with epilepsy who developed new focal seizures during adrenocorticotropic hormone therapy.

**Case presentation:**

The Japanese patient had posttraumatic epilepsy and developed intractable focal seizures at the age of 13 years. Adrenocorticotropic hormone therapy was administered when the patient was 14 years of age. On day 10 of treatment, he developed new focal seizures, which were characterized by left arm contractions followed by movements of touching things with his right hand and writhing and rocking his body left and right and back and forth as automatisms. The focal seizures clustered for 40 minutes and disappeared after suppository administration of 10 mg diazepam. These focal seizures did not reoccur after more than 2 years of follow-up.

**Conclusion:**

Adrenocorticotropic hormone-induced seizures can occur in children older than previously reported, and can occur in children with intractable seizures other than epileptic spasms.

## Background

Adrenocorticotropic hormone (ACTH) therapy for infantile spasms, including West syndrome, has been reported to potentially induce seizures [1–4]. These ACTH-induced seizures are characterized as new seizures that develop during ACTH therapy and cease after discontinuation of ACTH therapy. However, ACTH-induced seizures have not been described in patients with seizures other than epileptic spasms, patients without a history of epileptic spasms, and patients aged over 1 year.

Here, we report the findings of a school-aged child who developed ACTH-induced seizures. The findings presented in this case demonstrate that ACTH-induced seizures can occur in older patients with intractable seizures other than epileptic spasms.

## Case presentation

**Fig. 1:**
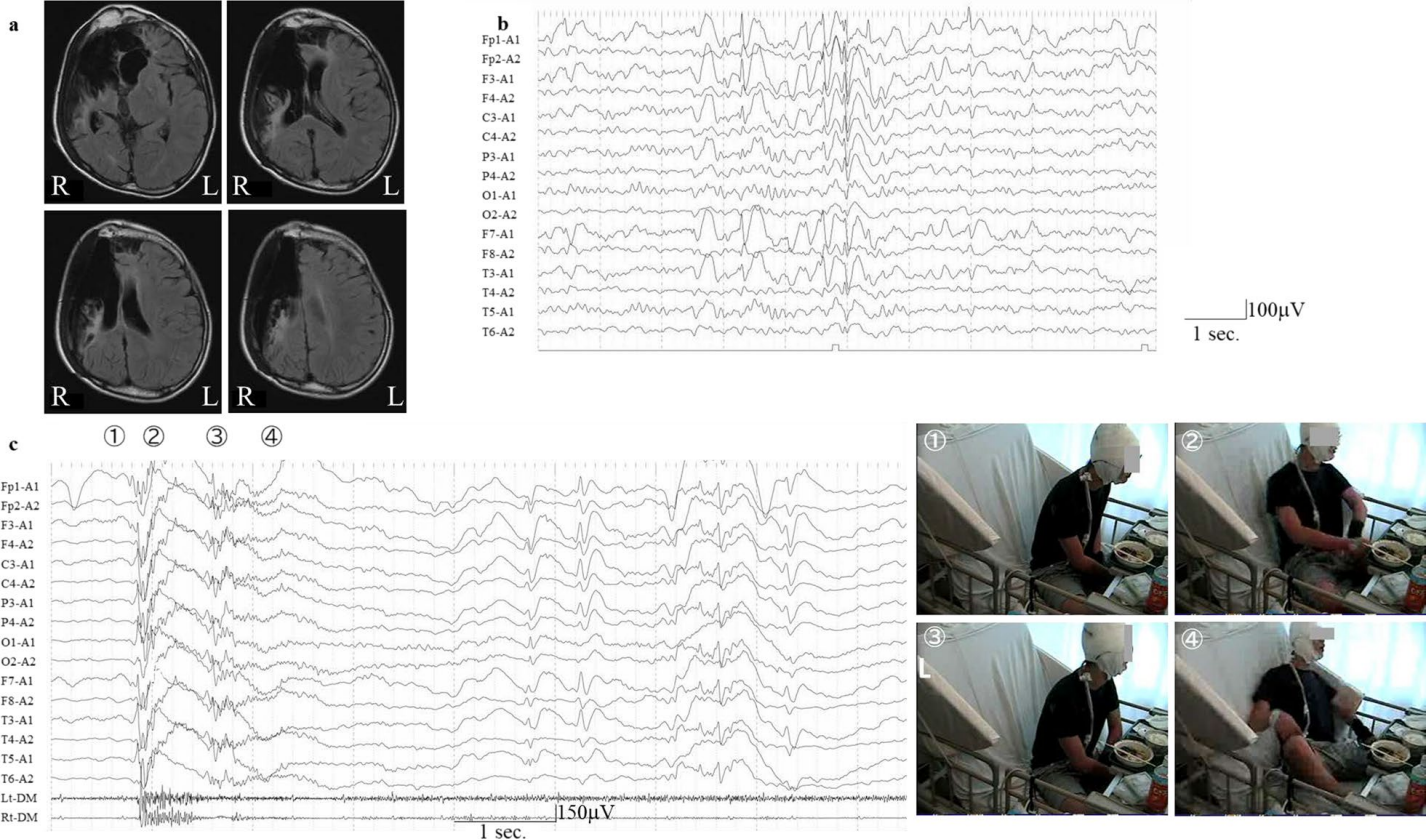
**a** Neuroradiological features. Partial damage to the right frontoparietal lobe and left frontal lobe on axial T2 fluid-attenuated inversion recovery imaging. L, left; R, right. **b** Interictal electroencephalography (EEG) before adrenocorticotropic hormone (ACTH) therapy. **c** Ictal EEG of a myoclonic seizure. An electromyogram of the deltoid muscle is overlaid at the bottom of this figure. Ictal video: ① Before the attack. ② His head began to turn to the left and left upper limb flexion associated with muscle contraction. ③ He began to fall backward. ④ He slumped against the back rest

**Fig. 2:**
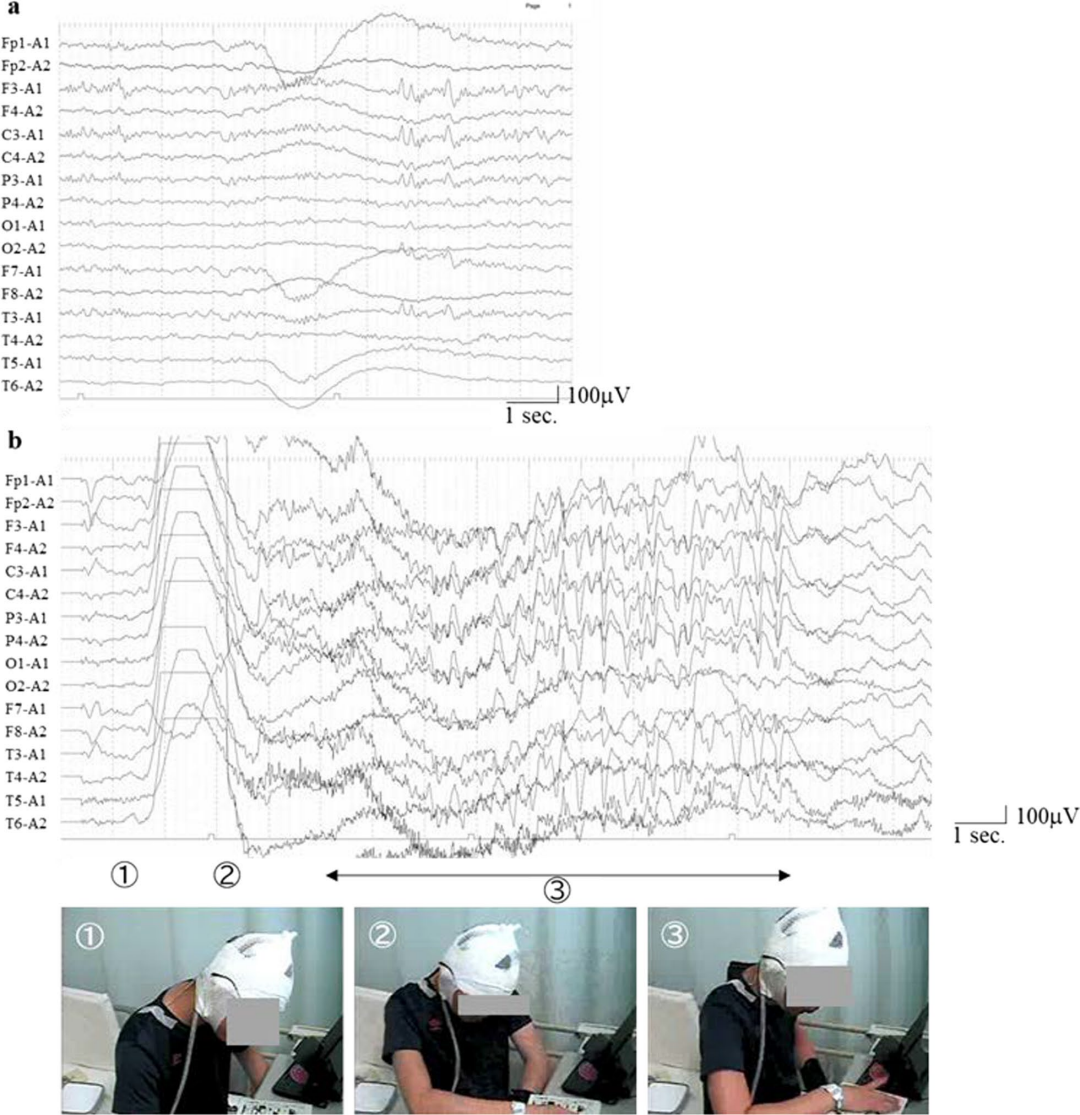
**a** Interictal electroencephalography (EEG) at 4 days after the development of adrenocorticotropic hormone (ACTH)-induced seizures. **b** Ictal EEG of a focal seizure during ACTH therapy. Ictal video: ① Before the attack. ② His left arm began to contract. ③ He began to touch things with his right hand and was writhing and rocking his body left and right, and back and forth

The patient was a 14-year-old Japanese boy who sustained a traumatic brain injury during a motor vehicle accident at the age of 6 years. The physical sequelae of this injury included partial damage to the right frontoparietal lobe and left frontal lobe, right enophthalmos, left hemiplegia, and loss of vision in the right eye (Fig. 1a). He developed unconsciousness following generalized seizures at the age of 10 years and was diagnosed with secondarily generalized tonic–clonic seizures. His seizures were not stopped by levetiracetam, sodium valproate, lamotrigine, clobazam, zonisamide, perampanel, phenobarbital, clonazepam, or lacosamide treatment. The frequency of seizures was recorded monthly. At 13 years of age, when carbamazepine was administered, brief muscle contractions of the upper limb occurred, and these seizures continued after carbamazepine was discontinued. Interictal electroencephalography (EEG) showed high-amplitude spikes or spikes and waves located in the left frontal pole to the frontal region (Fig. 1b). On video EEG monitoring, the ictal EEG revealed generalized rhythmic spike bursts preceded by a spike wave in the left hemisphere, followed by slow-wave bursts with left hemisphere dominance corresponding to his habitual seizures, which were characterized by turning his head to the left and left upper limb flexion along with muscle contraction in a sitting position after a fall backward and unawareness lasting 5 seconds (Fig. 1c). The seizures were diagnosed as focal seizures. In addition to the aforementioned drugs, oral administration of rufinamide and clorazepate did not reduce seizure frequency. After obtaining informed consent, ACTH therapy (synthetic ACTH, a zinc hydroxide suspension of tetracosactide acetate; Cortrosyn-Z [Daiichi-Sankyo, Japan]) was initiated at 10 months after video EEG monitoring. ACTH was administered as daily intramuscular injections (0.0125 mg/kg) for 2 weeks and was then gradually tapered off over 1 week. On day 10 of ACTH therapy, although his habitual seizures before ACTH therapy disappeared transiently, he developed new focal seizures characterized by left arm contractions followed by movements of touching things with his right hand and writhing and rocking his body left and right and back and forth as automatisms. On video EEG monitoring, the ictal EEG of the newly developing focal seizures showed generalized low-voltage fast waves followed by high voltage in the left hemisphere only, which was then followed by high-voltage slow-wave bursts with left hemisphere dominance (Fig. 2b). The focal seizures clustered for 40 minutes and disappeared after suppository administration of 10 mg diazepam. Interictal EEG at 4 days after the development of the new seizures showed spikes located in the left frontal region (Fig. 2a). These focal seizures did not reoccur after more than 2 years of follow-up. No new lesions were identified on brain computed tomography after the onset of focal seizures. On day 10 of ACTH therapy, his habitual seizures before ACTH therapy disappeared transiently; however, they reoccurred on day 14 after ACTH therapy and subsequently returned to daily frequency.

## Discussion and conclusion

As a paradoxical effect of ACTH therapy, new seizures can appear over the course of therapy, and are known as ACTH-induced seizures. In previous reports, patients with West syndrome were shown to develop ACTH-induced seizures; however, ACTH-induced seizures have not been previously reported in school-aged children. In the case described in this study, ictal EEG during the ACTH-induced seizure revealed a spike wave in the left hemisphere preceded by a burst of electromyographic activity followed by generalized rhythmic spike bursts. The patient’s ictal symptoms were characterized by turning his head to left and left upper limb flexion. Because the interictal and ictal EEG showed focal epileptiform discharges and ictal symptoms also showed lateralizing signs of motor symptoms, his seizures were diagnosed as focal motor seizures [5]. In previous studies, patients with West syndrome have been reported to develop ACTH-induced seizures; however, ACTH-induced seizures have not been previously reported in school-aged children with focal seizures (Table 1). Our findings suggest that ACTH-induced seizures can occur in school-aged children with epilepsy and focal seizures.

**Table 1:** The characteristics of seizures induced by adrenocorticotropic hormone

	Number of cases	Age/sex	Onset of ACTH-induced seizure after ACTH injection	Day of epileptic spasm cessation before or after ACTH discontinuous	Seizure type induced by ACTH	Drug during ACTH therapy	Ictal EEG of ACTH-induced seizure	Interictal EEG during appearance of ACTH-induced seizure
Kanayama *et al.* [1]	SWS = 1	7 mo/F	11th day	Day 15 after ACTH	Tonic seizure	none	15–20 c/s, 50–100-μV fast-wave bursts	N.D.
Otani *et al.* [2]	SWS = 1	5 mo/F	9th day	Day 14 after ACTH	Tonic seizure	PB, CZP	15–20 c/s, 50–100-μV fast-wave burst	Fast wave burst intermingled with Hyps.
Tokuyama *et al.* [3]	CWS = 1	5 mo/M	9th day	Day 7 after ACTH	Partial seizure	None	N.D.	N.D.
Fukui *et al.* [4]	CWS = 1	6 mo/M	10th day	Day 24 after ACTH	Partial seizure	VPA	P4 high-voltage slow → generalized	P3, P4 spikes
Our case	Posttraumatic epilepsy	14 y.o./M	10th day	Day 11 before ACTH	Partial seizure	RUF, CLZP, LTG	Generalized low-voltage fast-wave → left hemisphere → high-voltage slow	F3, C3 spikes

*CLZP* clorazepate, *C3* left central region, *CWS* cryptogenic West syndrome, *CZP* clonazepam, *EEG* electroencephalography, *F* female, *Hyps* hypsarrhythmia, *F3* left frontal region, *LTG* lamotrigine, *M* male, *mo* month, *ND* no data, *P3* left parietal region, *P4* right parietal region, *PB* phenobarbital, *RUF* rufinamide, *SWS* symptomatic West syndrome, *VPA* sodium valproate

As described in our previous report [4], the focal seizure in our case was suggested to be an ACTH-induced seizure for the following reasons: (1) the seizures, which were different from the patient’s habitual seizures, only appeared during ACTH therapy; (2) no new epileptic focus was revealed by EEG or brain imaging; (3) the seizures induced by ACTH therapy occurred on days 9–11 of consecutive ACTH injections (Table 1) [4]. In our case, the new seizures that occurred during ACTH therapy disappeared after a single dose of diazepam, suggesting that the seizures appeared only during ACTH therapy and were new epileptic seizures in the narrow sense. The findings suggest that ACTH-induced seizures can occur in school-aged children and in children with intractable seizures other than epileptic spasms.

The pathophysiology of ACTH-induced seizures remains unknown. Because ACTH-induced seizures developed in a patient with intractable posttraumatic epilepsy, these patients may share similar electrical and clinical characteristics. Both multifocal spikes on interictal EEG and multiple epileptic seizure types, which may reflect a hyperexcitable state and decreased seizure threshold, were found in these patients. Hypsarrhythmia has been observed in many patients with West syndrome, and these patients are likely to have other seizures. Among patients with infantile spasms, 39.1% have been reported to have partial seizures [6], and it is possible to transition from West syndrome to Lennox–Gastaut syndrome. One report suggested that patients with intractable posttraumatic epilepsy may have multiple epileptogenic foci because of poor surgical treatment results [7]. ACTH is a powerful therapeutic agent and may exert various central nervous system effects in infants and children. However, a state of hyperexcitability and a decreased seizure threshold may contribute to the development of ACTH-induced seizures.

In our case, ACTH therapy was used to treat intractable focal seizures. In a previous report, ACTH therapy was effective in older patients with intractable epilepsy other than spasms [8]. The author did not describe ACTH-induced seizures; therefore, their prevalence might not be high. However, ACTH-induced seizures may need to be considered when ACTH is used to treat these patients.

In conclusion, ACTH-induced seizures can occur in children older than previously reported, and can occur in children with intractable seizures other than epileptic spasms.

## Data Availability

Not applicable.

## Abbreviations

ACTH: Adrenocorticotropic hormone
EEG: Electroencephalography
